# Editorial: Language and Robotics

**DOI:** 10.3389/frobt.2021.674832

**Published:** 2021-04-12

**Authors:** Tadahiro Taniguchi, Takato Horii, Xavier Hinaut, Michael Spranger, Daichi Mochihashi, Takayuki Nagai

**Affiliations:** ^1^College of Information Science and Engineering, Ritsumeikan University, Kyoto, Japan; ^2^Graduate School of Engineering Science, Osaka University, Suita, Japan; ^3^Institut National de Recherche en Informatique et en Automatique (INRIA), Rocquencourt, Île-de-France, France; ^4^Sony Computer Science Laboratories, Tokyo, Japan; ^5^Department of Statistical Inference and Mathematics, The Institute of Statistical Mathematics, Tokyo, Japan

**Keywords:** language acquisition by robots, multimodal communication, concept formation, symbol grounding in robotics, symbol emergence in robotics, deep learning for robotics, emergence of communication

## 1. Introduction

Language in the real-world environment involves a wide range of challenges in robotics and artificial intelligence (AI). Service robots are required to communicate and collaborate with people using language in the real-world environment. When a robot receives a spoken command from a user in a domestic environment, the robot must understand its meaning in the context of the specific environment. For example, to understand the meaning of “please bring me a pen in Takato's room” the robot needs to know where to find a pen and where Takato's room is. Futhermore, words or expressions (i.e., sounds processed as symbols) can be invented naturally in our daily environment and their meaning can change (Spranger, 2016) over time (i.e., depending on the culture or age of the speaker). Robots thus need to adapt like humans to these versatile aspects of language and demonstrate the ability to learn any language (Hinaut and Twiefel, 2019). In robotics, language understanding inevitably involves multimodal learning, semantic mapping, and behavior learning. To enable a robot to interact orally with people in a long-term manner, we need to develop an AI that makes a robot learn and adapt to language in the real-world environment and in an on-line manner. This topic thus raises several challenges to bridge the gap from low-level sensorimotor interaction (Pagliarini et al., in press) to high-level compositional symbolic communication. Taking inspiration of how children acquire language can help to design the simplest mechanisms to deal with these challenges. Conversely, robotics can help modeling and test hypotheses about language acquisition and language grounding (Cangelosi and Schlesinger, 2015; Taniguchi et al., 2016, 2018; Hinaut and Spranger, 2019), in particular through cross-situational experiments (Taniguchi et al., 2017; Juven and Hinaut, 2020).

Following the successfully organized session “Language and Robotics” held in IEEE-IROS 2018^1^, we organized this Research Topic. We aimed to publish original papers from robotics, natural language processing, machine learning, and cognitive science to share knowledge about the state-of-the-art machine learning methods and perspectives that contribute to modeling language-related capabilities in robotics.

## 2. About the Research Topic

We are pleased to present five research articles related to semantic mapping, language understanding, motion segmentation, symbol emergence, and language evolution. In this section, we briefly introduce each paper.

First, three papers focused on language-related cognitive capabilities integrating real-world sensor information full of uncertainty and high-dimensional. Each method involves deep learning methods dealing with high-dimensional uncertain real data in robotics. Nagano et al. proposed a new machine learning method called a hierarchical Dirichlet process-variational autoencoder-Gaussian process-hidden semi-Markov model (HVGH). The method extended a hierarchical Dirichlet process-Gaussian process-hidden semi-Markov model (HDP-GP-HSMM) that can automatically segment time series data. HVGH integrated variational autoencoder and the HDP-GP-HSMM and achieved automatic motion segmentation along with representation learning. Katsumata et al. proposed a statistical semantic mapping method called SpCoMapping, which means spatial concept formation and semantic mapping. The proposed model employed Markov random field into a pre-existing spatial concept formation method and became able to learn the arbitrary shape of a place on a map. The method integrated multimodal information, e.g., language, vision, and position, to find semantic information of places. Tada et al. proposed a robust language understanding method by introducing noise injection into the sequence-to-sequence network. Recently, semantic parsing that enables a robot to understand the meaning of human user commands is developed based on deep learning methods. However, semantic parsing in natural language processing does not assume the existence of speech recognition errors. This paper showed the conventional idea of noise injection to sequence-to-sequence network semantic parsing can improve the robustness of a robot's language understanding.

Second, two papers focused on the emergence, or evolution, of symbols. Cambier et al. described the perspectives of language evolution in swarm robotics. They advocated an approach based on language games for the further development of emergent communication in swarm robots. They suggested that swarm robotics can be an ideal testbed to advance research on the emergence of language-like communication. Hagiwara et al. proposed a new computational model representing symbol emergence. The model proposed in this paper regarded symbol emergence as a multiagent multimodal categorization problem. The convergence of the algorithm was guaranteed based on the theory of Markov chain Monte Carlo. This symbol emergence model involved sharing signs among agents and making each agent form internal representations based on its sensorimotor information.

## 3. Next Step

With the great success of this Research Topic, we organized related workshops and a tutorial^2^. A survey paper related to this topic has already been published (Tangiuchi et al., 2019). We believe that integrating low-level and high-level cognitive capabilities (Nakamura et al., 2018; Taniguchi et al., 2020) in conjunction with language learning in the real-world environment is crucial to creating an artificial cognitive system, i.e., a robot, which can conduct lifelong learning in the real-world environment and achieves long-term human-robot interaction to support daily human activities. The intersection of language and robotics is a crucial Research Topic for further advancement in robotics and AI. We hope that this special issue will accelerate the cutting-edge studies in robotics and AI that aim to create human-level embodied AI that can communicate and collaborate with people in the real-world environment.

## Author Contributions

All authors listed have made a substantial, direct and intellectual contribution to the work, and approved it for publication.

## Conflict of Interest

The authors declare that the research was conducted in the absence of any commercial or financial relationships that could be construed as a potential conflict of interest.

## Footnotes

^1^The Workshop on Language and Robotics: http://iros2018.emergent-symbol.systems/home.

^2^A first workshop was on Deep Probabilistic Generative Models for Cognitive Architecture in Robotics: https://sites.google.com/site/dpgmcar2019/. A second workshop was on Sensorimotor Interaction, Language and Embodiment of Symbols (SMILES): https://sites.google.com/view/smiles-workshop/. The tutorial was on Deep Probabilistic Generative Models for Robotics: https://sites.google.com/view/dpgmfr/home.

## References

[B1] Cangelosi A. Schlesinger M. (2015). Developmental Robotics: From Babies to Robots. Cambridge, MA: MIT Press.

[B2] Hinaut X. Spranger M. (2019). Learning to parse grounded language using reservoir computing, in 2019 Joint IEEE 9th International Conference on Development and Learning and Epigenetic Robotics (ICDL-EpiRob) (Oslo: IEEE), 266–271.

[B3] Hinaut X. Twiefel J. (2019). Teach your robot your language! trainable neural parser for modeling human sentence processing: Examples for 15 languages. IEEE Trans. Cogn. Dev. Syst. 12, 179–188. 10.1109/TCDS.2019.2957006

[B4] Juven A. Hinaut X. (2020). Cross-situational learning with reservoir computing for language acquisition modelling, in 2020 International Joint Conference on Neural Networks (IJCNN) (Glasgow: IEEE), 1–8.

[B5] Nakamura T. Nagai T. Taniguchi T. (2018). Serket: an architecture for connecting stochastic models to realize a large-scale cognitive model. Front. Neurorobot. 12:25. 10.3389/fnbot.2018.00025 29997493 PMC6028621

[B6] Pagliarini S. Leblois A. Hinaut X. (in press). Vocal imitation in sensorimotor learning models: a comparative review. IEEE Trans. Cogn. Dev. Syst. 10.1109/TCDS.2020.3041179

[B7] Spranger M. (2016). The Evolution of Grounded Spatial Language. Language Science Press (Berlin).

[B8] Tangiuchi T. Mochihashi D. Nagai T. Uchida S. Inoue N. Kobayashi I.. (2019). Survey on frontiers of language and robotics. Adv. Robot. 33, 700–730. 10.1080/01691864.2019.1632223

[B9] Taniguchi A. Taniguchi T. Cangelosi A. (2017). Cross-situational learning with bayesian generative models for multimodal category and word learning in robots. Front. Neurorobot. 11:66. 10.3389/fnbot.2017.00066 29311888 PMC5742219

[B10] Taniguchi T. Nagai T. Nakamura T. Iwahashi N. Ogata T. Asoh H. (2016). Symbol emergence in robotics: a survey. Adv. Robot. 30, 706–728. 10.1080/01691864.2016.1164622

[B11] Taniguchi T. Nakamura T. Suzuki M. Kuniyasu R. Hayashi K. Taniguchi A.. (2020). Neuro-serket: development of integrative cognitive system through the composition of deep probabilistic generative models, in New Generation Computing (Cham), 1–26.

[B12] Taniguchi T. Ugur E. Hoffmann M. Jamone L. Nagai T. Rosman B.. (2018). Symbol emergence in cognitive developmental systems: a survey. IEEE Trans. Cogn. Dev. Syst. 11, 494–516. 10.1109/TCDS.2018.2867772

